# Analytic Gradients for Density Fitting MP2 Using Natural Auxiliary Functions

**DOI:** 10.1021/acs.jpca.4c02822

**Published:** 2024-07-29

**Authors:** Klára Petrov, József Csóka, Mihály Kállay

**Affiliations:** † Department of Physical Chemistry and Materials Science, Faculty of Chemical Technology and Biotechnology, Budapest University of Technology and Economics, Műegyetem rkp. 3., H-1111 Budapest, Hungary; ‡ HUN-REN−BME Quantum Chemistry Research Group, Műegyetem rkp. 3., H-1111 Budapest, Hungary; § MTA−BME Lendület Quantum Chemistry Research Group, Műegyetem rkp. 3., H-1111 Budapest, Hungary

## Abstract

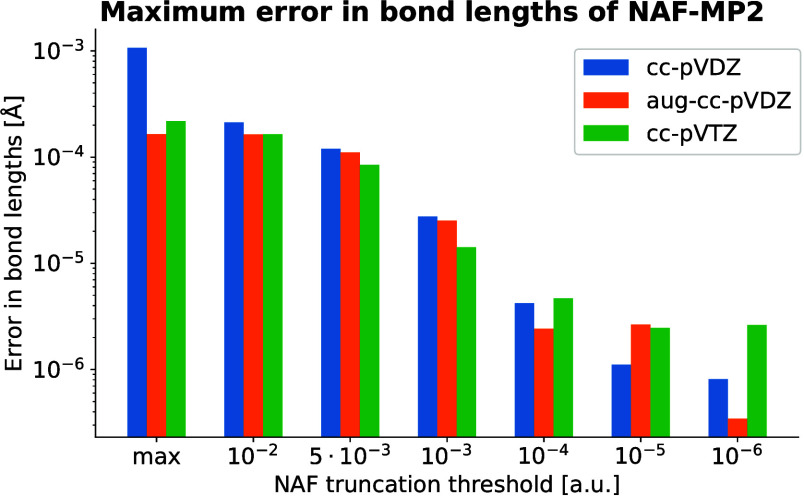

The natural auxiliary function (NAF) approach is an approximation to decrease the size of the auxiliary basis set required for quantum chemical calculations utilizing the density fitting technique. It has been proven efficient to speed up various correlation models, such as second-order Møller–Plesset (MP2) theory and coupled-cluster methods. Here, for the first time, we discuss the theory of analytic derivatives for correlation methods employing the NAF approximation on the example of MP2. A detailed algorithm for the gradient calculation with the NAF approximation is proposed in the framework of the method of Lagrange multipliers. To assess the effect of the NAF approximation on gradients and optimized geometric parameters, a series of benchmark calculations on small and medium-sized systems was performed. Our results demonstrate that, for MP2, sufficiently accurate gradients and geometries can be achieved with a moderate time reduction of 15–20% for both small and medium-sized molecules.

## Introduction

The calculation of accurate gradients with respect to nuclear displacements and thus to predict molecular equilibrium structures is a fundamental yet computationally demanding task in quantum chemistry. The development of efficient analytic gradients for the second-order Møller–Plesset (MP2) approach,^1^ as one of the simplest post-Hartree–Fock (HF) electron correlation methods, is a substantial step toward the implementation of gradients for more sophisticated approaches like the coupled-cluster (CC) method.

The capabilities and limitations of MP2, along with its energy gradients, have been extensively explored and are well-understood.^2^ In the past decades, tremendous efforts have also been undertaken to reduce its steep computational scaling [, where *N* is a measure of the system size] of MP2 energies and gradients. These developments follow several strategies. On one hand, the evaluation and transformation of electron repulsion integrals (ERIs) were sped up by factorizing four-index ERIs with the density fitting method (DF) also referred to as resolution of the identity method (RI),^3,4^ Cholesky decomposition (CD),^5^ tensor hyper-contraction (THC),^6^ the pseudospectral approach to ERIs,^7,8^ or by mimicking a larger basis set in the self-consistent field (SCF) calculation using the dual basis method.^9,10^ On the other hand, the locality of electron correlation was utilized employing molecular orbitals (MOs) other than the canonical ones. These developments include the atomic orbital (AO) based MP2 approaches,^11−13^ which usually utilize Laplace transform,^14,15^ and the various localized MO-based methods, which either solve the MP2 equations for the entire system at the same time^16−21^ or partition the molecular problem in smaller parts and evaluate the contribution of the fragments separately.^22−26^ The refinement of these techniques eventuated even linear-scaling methods.^26−33^

Besides the cost reduction of MP2, additional efforts were also taken to improve the accuracy of the method by various approaches. The most important developments along this line include the spin-scaled MP2 approaches, which improve the correlation energy by separate scaling of the Coulomb- and the exchange-like terms,^34,35^ dispersion-corrected MP2, where the long-range behavior of MP2 is corrected,^36^ orbital-optimized MP2, where the MP2 energy is made stationary with respect to the rotation of the MOs,^37,38^ and explicitly correlated MP2, where the wave function explicitly includes the interelectronic distances in the form of pair functions.^39^ These improved MP2 models were also combined with the aforementioned cost-reduction approximations resulting in low- or linear-scaling methods.^40−43^

The theory and implementation of MP2 analytic gradients and second derivatives are also well established. Analytic derivatives for conventional MP2 have been available for a long time.^44−48^ Of the above reduced-cost approaches, analytic gradients have been implemented for DF-MP2,^49−53^ CD-MP2,^54^ THC-MP2,^6,55^ the dual-basis MP2,^56^ AO-MP2,^57^ divide and conquer MP2,^58^ and for various local MP2 methods.^59−66^ Analytic gradients are also available for spin-scaled and explicitly correlated MP2 even in conjunction with the considered cost-reduction techniques.^67−70^

From the point of view of our study, the most important approach to reduce the prohibitively high computational cost of MP2 energy and gradient calculations involves the use of the DF approximation. As the major time-consuming factors in conventional MP2 calculations correspond to the evaluation of two-electron four-center ERIs and their subsequent transformations, calculations can be efficiently reduced by factorizing four-index ERIs into three-index and two-index integrals with the help of DF. In the DF approach, the ERIs are regarded as Coulomb integrals of generalized electron densities, and these density distributions are fitted with a linear combination of an auxiliary basis, which consists of atom-centered Gaussian functions in most applications.^71−74^ The largest efficiency of factorizing two-electron ERIs is gained when the residual function is minimized using the Coulomb metric. Although DF successfully reduces the computational prefactor associated with MP2 computations, it leaves the underlying fifth-order scaling untouched. However, time savings of DF-MP2 can be 1–2 orders of magnitude compared to the costs of a conventional MP2 calculations, especially if DF is combined with other cost reduction techniques.^27,28,75^

Calculations utilizing DF can further be accelerated by our natural auxiliary function (NAF) approach,^76^ which efficiently reduces the size of the auxiliary basis set. In the NAF approximation, a partial singular value decomposition (SVD) is carried out on three-center Coulomb integrals. Keeping singular vectors corresponding to the largest singular values results in a fitting basis with which a new, reduced-sized auxiliary basis set can be defined. The NAF approximation was successfully utilized to speed up the MP2,^26,76^ the direct random-phase approximation,^76^ the CC singles, doubles with perturbative triples [CCSD(T)],^77^ and various excited-state and ionization methods^78−81^ as well as explicitly correlated MP2^82^ and CCSD(T).^83^ The NAF approach turned out to be useful for local correlation methods^26,84,85^ and also for the automatic generation of auxiliary basis sets.^86^

The purpose of this work is to lay down the foundations of the theory of analytic derivatives for correlation methods employing the NAF approximation. We present the required conditions to be considered in a Lagrangian formulation of energy derivatives and derive the analytical gradients of the NAF-MP2 energy. We discuss the modifications required in the DF-MP2 algorithm to implement NAF-MP2 energy gradients. We also determine the appropriate truncation threshold for the NAFs and demonstrate the applicability of our approach by scrutinizing the accuracy and speedups achieved for a few selected examples.

## Methods

In what follows, first, we briefly outline the NAF technique. Then, the necessary modifications of the DF-MP2 Lagrangian and gradient expression are discussed. Finally, we comment on the algorithm and present the details of the implementation. A restricted closed-shell formalism with the frozen-core approximation is used throughout the paper, and our notation conventions for index symbols are listed in Table 1.

**Table 1:** Notation conventions for the various orbital spaces and for the number of the corresponding orbitals

symbol	number	definition
μ, ν	*n*_AO_	AO
*p*, *q*	-	general MO
*f*	-	frozen core MO
*i*, *j*, *k*	*n*_occ_	correlated occupied MO
*a*, *b*, *c*	*n*_virt_	virtual MO
*P*, *Q*, *R*, *S*	*n*_aux_	auxiliary basis function
, , ,	*n*_NAF_	kept NAF
, , ,	*n*_NAF′_	dropped NAF

### Natural Auxiliary Functions

In the DF approximation with Coulomb metric, four-center ERIs are expanded in an auxiliary basis as
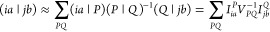
1where (*ia*|*jb*) stands for four-center ERIs in the MO basis using Mulliken’s notation, while  and *V*_*PQ*_ = (*P*|*Q*) denote the three-center and two-center ERIs, respectively. The inverse of the two-center ERIs is usually factorized. One of the most efficient methods is the CD of the two-center integral matrix:
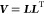
2where ***L*** is a lower triangular matrix. However, the forthcoming discussion is valid for any other factorization of the inverse two-center integral matrix.

The cost of the reconstruction of the four-center ERIs from the arising ***J*** = ***IL***^–T^ matrix is proportional to the size of the auxiliary fitting basis set (*n*_aux_). Thus, the reduction of the size of the auxiliary basis is desirable without the decrease of accuracy. This reduction can, in principle, be achieved by the SVD of matrix ***J*** as ***J*** = ***M***Σ***N***^T^, where ***M*** and ***N*** are unitary and contain the left and right singular vectors of ***J***, respectively, while **Σ** is diagonal holding the singular values. The SVD is used to reduce the rank of a matrix by keeping the *r* largest singular values and the corresponding singular vectors. This way, the truncated matrix is the best rank *r* approximation to the matrix in the Frobenius norm.

Instead of a full SVD, the singular values and the right singular vectors of matrix ***J*** can be efficiently computed by diagonalizing the following matrix:^76^
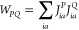
3The eigenvalues of ***W*** equal the squares of the singular values, whereas its eigenvectors are the right singular vectors. These eigenvectors, residing in the columns of ***N***, called the NAFs. The auxiliary index of the three-center Coulomb integrals ***J*** can be transformed with ***N*** to the new auxiliary fitting basis dubbed as the NAF basis:
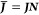
4

Keeping only the most important *n*_NAF_ number of eigenvectors with eigenvalues greater than a threshold, ε_NAF_, a reduced-dimension  matrix can be defined as , where overlined capital indexes  labels the kept NAFs. This  matrix is the best rank *n*_NAF_ approximation of ***J*** in the least-squares sense. However, as we will see, at the calculation of energy gradients, the use of the *n*_NAF′_ = *n*_aux_ – *n*_NAF_ number of dropped eigenvectors of ***W*** is also necessary: , where column  of matrix ***N*** contains the eigenvectors of ***W*** with eigenvalues smaller than ε_NAF_.

Note that because of algorithmic considerations, matrix ***W***′ = ***I***^*T*^***I*** is calculated directly from the three-center ERIs in the MO basis. The fitting with the inverse Cholesky matrix is performed on matrix ***W***′ as ***W*** = ***L***^–1^***W***′***L***^–T^. The three-center ERIs in the NAF basis are then calculated as , where ***N***′ = ***L***^–T^***N***.

### The Energy and the Lagrangian

To calculate the total energy in MP2 theory, we have to add the HF energy and the MP2 correlation energy together:
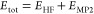
5The former, supposing a restricted closed-shell approach, reads as
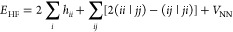
6where *h*_*ii*_ is the corresponding element of the core Hamiltonian, and *V*_NN_ stands for the nuclear repulsion energy. We suppose that the DF approximation is also utilized at the HF level with an auxiliary basis distinct from the one used in the correlation calculation. The MP2 correlation energy is defined as
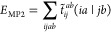
7where  stands for a contravariant MP2 amplitude:
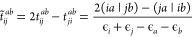
8with ϵ_*q*_ as the canonical HF molecular orbital energy and  as an MP2 amplitude:
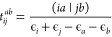
9Using three-center ERIs in the truncated NAF basis to approximate the four-center ERIs in eqs 7 to 9, the latter can be expressed as
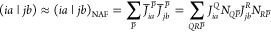
10

The MP2 energy, even without the NAF approximation, is nonvariational, thus the method of Lagrange multipliers is invoked to calculate the analytic derivatives. The following Lagrangian can be delineated for our problem:
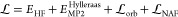
11The second term on the right side stands for the MP2 Hylleraas functional,^16,47,87^ which makes the Lagrangian stationary with respect to the MP2 amplitudes. The Hylleraas functional in terms of NAF-approximated quantities takes the form
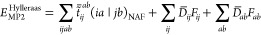
12where  and  are the symmetric MP2 densities, and *F*_*pq*_ stands for the respective Fock matrix element. Here, the overline above a quantity signs that the four-center ERIs in it are calculated in the NAF approximation.

The third term in Lagrangian eq 11 expresses the response of the system to the change in the HF reference MOs, that is, the orbital relaxation effect, and also accounts for the core–valence separation if the frozen core approximation was applied:
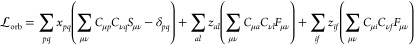
13Here, *C*_*μp*_ denotes an MO coefficient, *S*_*μν*_ stands for the overlap matrix in the AO basis, while ***x*** and ***z*** hold the Lagrange multipliers. The first term of this expression is the Lagrangian constraint corresponding to the orthogonality condition of the MOs, where *x*_*pq*_ and *x*_*qp*_ are not independent, thus we impose that ***x*** is symmetric. The second term in eq 13 expresses the Brillouin condition, which prevents mixing of occupied (core or valence) and virtual orbitals. The third term stands for the core–valence separation condition.

The last term in eq 11 corresponds to the NAF approximation used for the four-center ERIs and is common to all methods that employ NAFs. We introduce two new constraints as
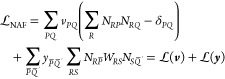
14The first one expresses the orthogonality of NAFs, with  number of independent multipliers in the symmetric matrix ***v***. The second term contains the eigenvalue problem of ***W*** that guarantees that the kept-dropped  block of the eigentransformed ***W*** matrix is zero. Note that the energy is invariant to unitary transformations to the kept–kept and dropped–dropped blocks, therefore it is not necessary to assign Lagrange multipliers to these blocks. The *n*_NAF_*n*_NAF′_ number of independent multipliers corresponding to this condition are collected in ***y***.

The Lagrangian has to be stationary with respect to all parameters in it:
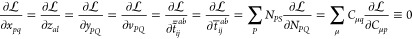
15The first six conditions are already fulfilled by the construction of the Lagrangian. From the next stationarity condition, we can calculate Lagrange multipliers ***v*** and ***y***. The Hylleraas energy functional depends only on the kept eigenvectors in ***N***, thus we have
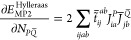
16while the  part of the Lagrangian must be differentiated with respect to all elements in ***N***:
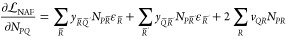
17where ε_*P*_ is the eigenvalue corresponding to the *P*th eigenvector of ***W***, located in the *P*th column of ***N***. Inserting partial derivatives 16 and 17 into the stationary condition, we get:
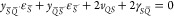
18where we introduced . Simple algebraic operations lead immediately to Lagrange multipliers ***y*** and ***v***:
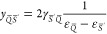
19
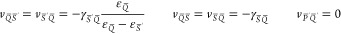
20

The last stationarity condition in eq 15 refers to the rotation of the MOs and allows us to calculate Lagrange multipliers ***x*** and ***z***. All terms of Lagrangian eq 11 except  contribute to this expression:
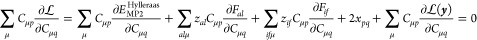
21Assuming that ***x*** is symmetric, we solve the antisymmetric part of eq 21. The first, the third, and the last terms add contribution to the core–valence equations and eventuate multipliers *z*_*if*_:
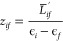
22The remaining terms result in the well-known Z-vector solution of the coupled-perturbed HF (CPHF) equations:^60,88^
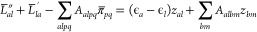
23where we have introduced composite matrix , *A*_*pqrs*_ = 4(*pq*|*rs*) – (*ps*|*rq*) – (*pr*|*sq*), and . The  and  quantities are similar to the corresponding standard DF-MP2 quantities;^49^ however, they include derivatives not only from the first term of the Hylleraas functional but also from the derivative of :
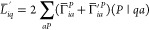
24
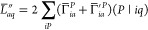
25where
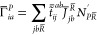
26
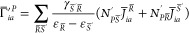
27Solution of the Z-vector equation gives the occupied-virtual block of the relaxed MP2 density matrix, denoted here by multipliers *z*_*al*_. Substituting back all known multipliers into the stationarity condition in eq 21, we can get multipliers ***x***.

### Calculation of the Gradient

To calculate the final gradient, the Lagrange functional has to be differentiated with respect to external perturbation ξ:
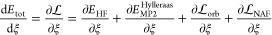
28The derivative of the first term of the Hylleraas functional can be expressed in the usual way as
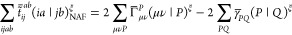
29where (*μν*|*P*) denotes the three-center ERIs in the AO basis, and superscript ξ indicates that the integral is differentiated with respect to ξ.  is calculated similarly as in the conventional DF-MP2 method^49^ but contains the three-center ERIs in the NAF basis. For , we obtain
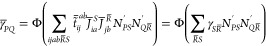
30where we have introduced function Φ:
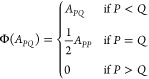
31The appearance of Φ is a consequence of the derivative of the Cholesky matrix,^89^ which can be expressed as
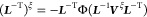
32Note that in , all three-center ERIs in the NAF basis are used , not only the kept ones .

The last term of the Lagrangian derivative eq 28 can also be expressed with the two- and three-center AO integrals, giving corrections to matrices  and :
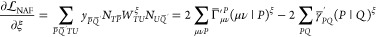
33where , while  is
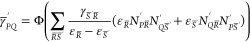
34

The rest of the derivative Lagrangian (eq 28) is evaluated in the usual way,^49^ resulting in the final expression for the energy derivative:
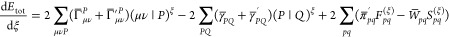
35where , , , and  is an element of the Fock matrix that is built from derivative integrals.

### Algorithmic Considerations

The general algorithm for the present NAF-MP2 approach is based on the DF-MP2 method^49^ implemented in the MRCC program,^90^ which is structured as follows:

1. Solve HF equations
2.
  (a) Construct three-center ERIs
  (b) Assemble four-center ERIs, calculate MP2 energy, symmetric MP2 densities, and intermediate
  (c) Calculate **Γ**, **γ**, ***L***′, and ***L***″ matrices
3. Solve Z-vector equation
4. Calculate MP2-level properties

**Figure 1:**
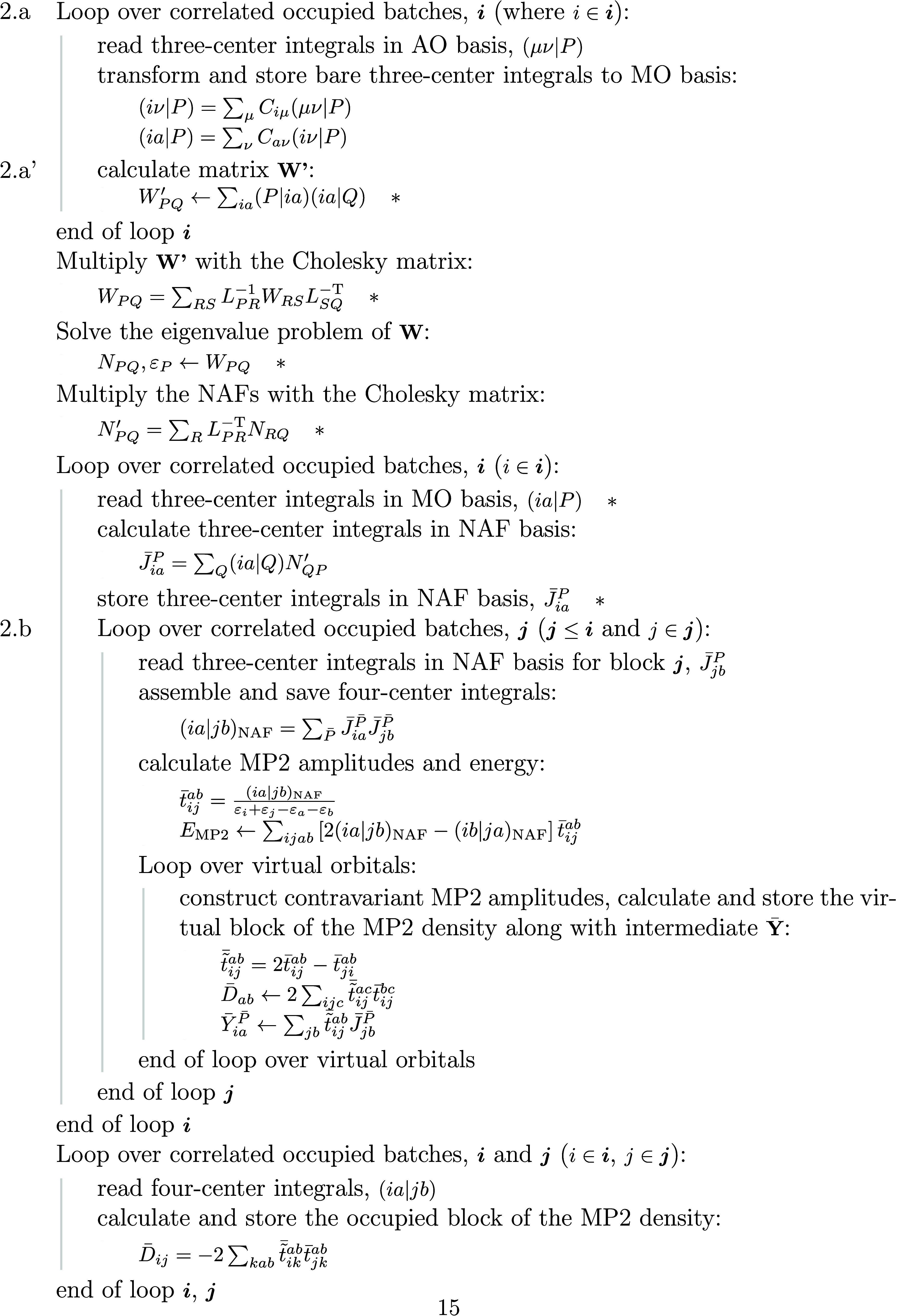
Algorithm of NAF-MP2 first derivatives: steps 2.a, a’, and b.

**Figure 2:**
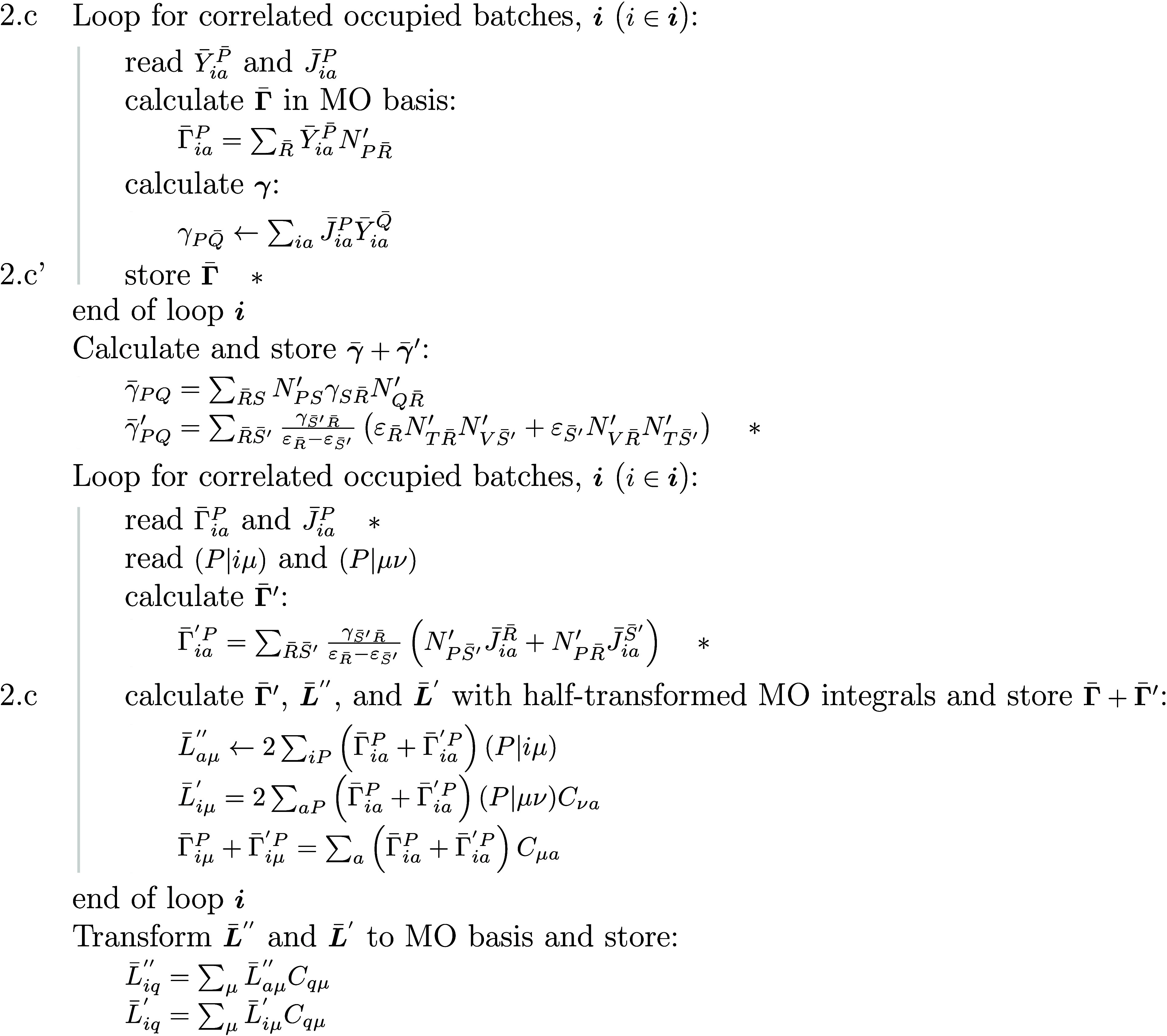
Algorithm of NAF-MP2 first derivatives: steps 2.c and c’.

The NAF-MP2 workflow only differs from the standard DF-MP2 code in step 2. The detailed algorithm of this step for NAF-MP2 is shown in Figure 1 and Figure 2.

In this algorithmic scheme, steps 2.a’ and 2.c’ feature the main differences between the standard DF-MP2 and NAF-MP2 codes, namely the calculation of the three-center ERIs in the NAF basis and the corrections to  and  originating from the introduction of the NAFs into the Lagrangian. Each step that means additional operation in the NAF-MP2 algorithm compared to DF-MP2 is signed by an asterisk, and the corresponding operation counts are summarized in Table 2. Another substantial difference in the algorithms is that DF-MP2 performs steps 2.a and 2.b in one loop for subsets of correlated occupied orbitals, whereas the NAF-MP2 method divides these steps in two separate loops with the construction of the NAFs in between (2.a’).

**Table 2:**
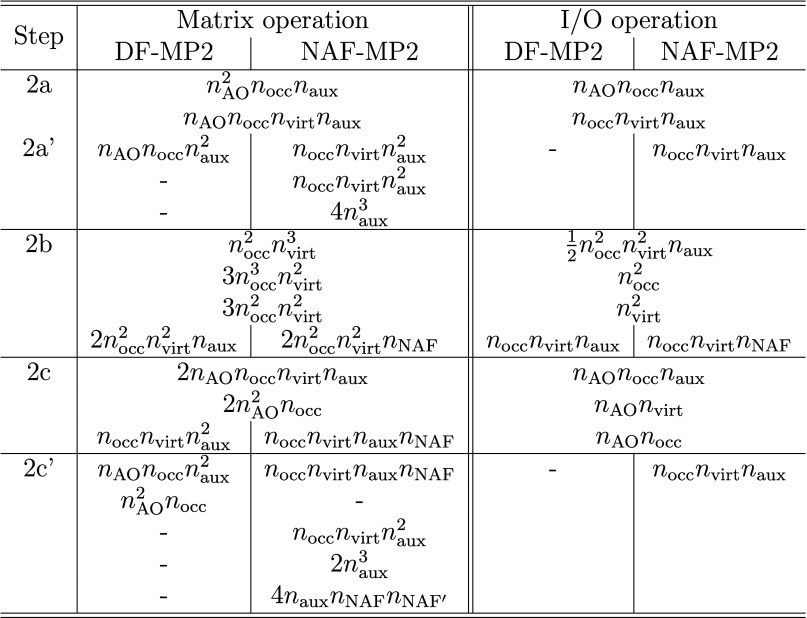
Summary of the matrix and I/O operation counts in various steps of the algorithm for DF-MP2 and NAF-MP2

Going through the differences in the algorithms step by step, 2.a’ contains operations to calculate the NAFs and to transform the three-center ERIs into the NAF basis. The construction of the NAFs requires approximately  additional floating point operations and only the storage of matrix ***W*** in the memory compared to the parent DF-MP2 method. The transformation of the integrals to the NAF basis requires approximately the same number of operations as the fitting of the bare three-center integrals with the Cholesky matrix in the original DF-MP2 algorithm. However, the reading of the three-center AO integrals and the storage of the three-center ERIs in the NAF basis mean additional I/O operations and mass storage requirements on the scale of *n*_occ_*n*_virt_*n*_aux_.

Step 2.b comprises the computational bottlenecks of the analytic calculation of first-order properties besides the solution of the Z-vector equation (step 3). These include the calculation of the symmetric MP2 densities along with the assembly of the four-center ERIs and the calculation of intermediate . Though in principle this point is not modified compared to the DF-MP2 algorithm, the number of operations strongly depends on the number of auxiliary functions. While the calculation of the densities as well as the solution of the Z-vector equations are independent of the number of auxiliary functions used in the correlation calculation, thus are left untouched by the NAF approach, the construction of the four-center ERIs and intermediate ***Y*** scale as  in the genuine DF-MP2 algorithm but only as  in NAF-MP2. The reduction of the operation count of these fifth-power scaling operations by a factor of *n*_NAF_/*n*_aux_ is the main advantage of the NAF-MP2 method. A minor gain of this step is that the size (thus the storage requirement) of matrix  is also reduced. Note that the calculation of the occupied block of the symmetric density matrix implies the storage of  matrix elements in both the DF-MP2 and the NAF-MP2 algorithms, which means the main mass storage requirement.

In step 2.c, the reduced-size  cuts down the operation count of the matrix multiplications and the I/O operation requirement by a factor of *n*_NAF_/*n*_aux_. Step 2.c’ comprises additional matrix operations for the calculation of  and  on the scale of  + , while the rate of the fourth-power scaling terms decreases slightly from  to *n*_occ_*n*_virt_*n*_aux_*n*_NAF_. This step also requires the additional storage of matrix elements  and implies further I/O operations for this quantity.

### Computational Details

The analytic gradients for the NAF-MP2 method have been implemented in the MRCC quantum chemistry program.^90^

All calculations were carried out with Dunning’s correlation consistent double-ζ, triple-ζ, and double-ζ augmented with diffuse functions basis sets, cc-pVDZ, cc-pVTZ, and aug-cc-pVDZ, respectively.^91−93^ The DF approximation was applied to both the SCF and the MP2 calculations using the auxiliary basis sets (aug-)cc-pVXZ-RI-JK^94^ and (aug-)cc-pVXZ-RI,^95,96^ respectively. The frozen core approximation was employed in the post-HF calculations.

The performance of our method was compared to conventional DF-MP2 method implemented in MRCC. The accuracy of our NAF-MP2 implementation was tested on the gradients and the optimized geometric parameters of 30 molecules originally suggested by Baker.^97^ The speed-ups attained were evaluated for molecules of various size: penicillin,^98^ androstendione,^99^ a DNA fragment containing an adenine-thymine base pair (DNA1),^13^ indinavir.^100^ The number of atoms, electrons, AOs, fitting functions and the computation times for our test systems are given in the Supporting Information (SI). The reported computation times are wall-clock times measured on a single 6-core Intel Xeon E5–1650 CPU with 128 GB of RAM. We did not use hyperthreading, the number of OpenMP threads equals the number of the physical cores.

## Results and Discussion

### The Accuracy of NAF-MP2 Gradient

Since the operation counts for two of the rate-determining steps of the NAF-MP2 algorithm, detailed in section Algorithmic considerations, are proportional to the number of retained NAFs, it is crucial to keep *n*_NAF_ as low as possible while introducing negligible error. Thus, we have to find the optimal threshold for the eigenvalues of matrix ***W***, ε_NAF_, which determines the number of kept NAFs. In our algorithm, we do not let *n*_NAF_ to be lower than the number of the AO basis functions, *n*_AO_, because too small fitting bases may result in unphysical two-electron integrals even if the energies are meaningful due to error compensation. The value of ε_NAF_ that belongs to *n*_NAF_ = *n*_AO_ will be referred to as its maximum (max) value.

**Figure 3:**
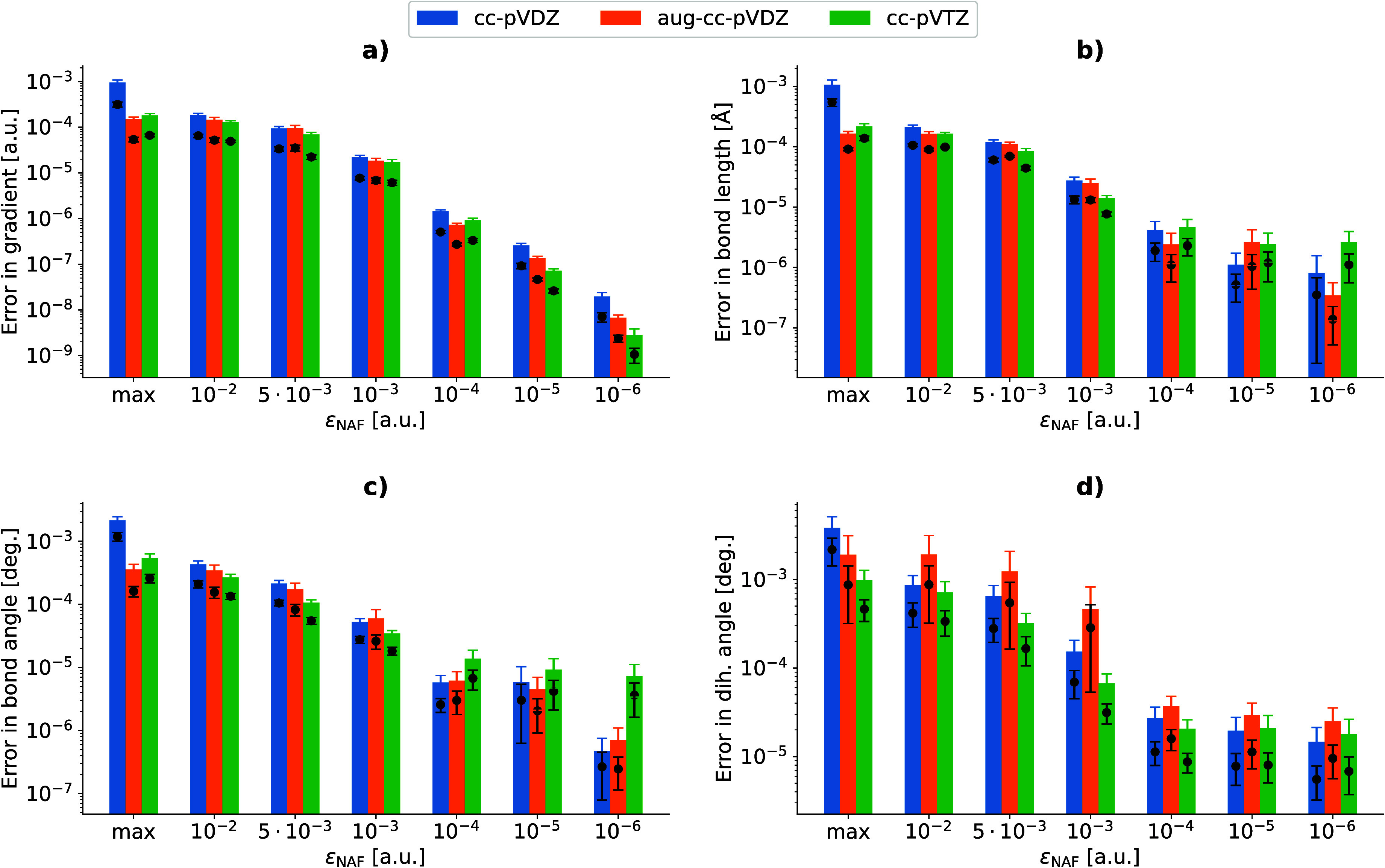
Error of NAF-MP2 gradients (a) and optimized geometric parameters (b, c, d) as a function of ε_NAF_ for Baker’s test set with three different basis sets. The bars and their errors correspond to the mean and the standard deviation of the maximum errors, respectively, while the dots and their errors represent the mean and standard deviation of rms errors, respectively.

First, we performed test calculations for 30 small molecules collected in Baker’s test set. The gradient components of the molecules at the nonoptimized geometries were compared to standard DF-MP2 results as a function of ε_NAF_ calculated with three different basis sets. The evaluated error statistics include the maximum (max) error and the root-mean-square (rms) error of the gradient components. The results are shown in panel a of Figure 3. We note that the analytical gradient was verified against the numerical gradient for three small molecules with ε_NAF_ = 10^–2^ a.u.: neopentane, naphthalene, and hydrazobenzene. In all cases, the maximum error of the analytical gradient was found to be less than 10^–7^ a.u.

The plot shows that the errors in the gradients are already around 10^–4^ a.u. for ε_NAF_ values ranging from the maximum to 10^–3^ a.u. The only exception is the cc-pVDZ basis set with maximal ε_NAF_, where the gradient error rises an order of magnitude. The gradient errors drop below 10^–5^ a.u. for ε_NAF_ < 10^–3^ a.u. and show a continuous lowering with decreasing ε_NAF_.

Geometry optimizations were also carried out for the molecules of Baker’s test set. The mean and standard deviation of max and rms errors for the optimized geometric parameters (bond lengths, bond angles, and dihedral angles) in comparison to standard DF-MP2 results are displayed in panels b, c, and d of Figure 3. These plots show the very same trends as those for the gradient errors. Sufficiently optimized geometries can be achieved even with the highest threshold values, however, the use of maximal ε_NAF_ with the cc-pVDZ basis set introduces relatively large but still negligible errors in the geometries of several molecules.

Table 3 shows the average percentage of the kept NAFs for the above-discussed geometry optimizations. The standard deviation of these values is below 1% showing that ε_NAF_ defines well the relative number of retained NAFs. Moreover, the number of the retained NAFs changes by at most 0.2% during the geometry optimization steps for the molecules of Baker’s test set. The selected ε_NAF_ thresholds provide a good screening of the NAFs. For the cc-pVTZ basis set, the percentage of the retained NAFs is consistently larger than with the double-ζ basis sets. The difference between the latter with and without diffuse functions is moderate. The values show a larger discrepancy only in the case of the maximal ε_NAF_ threshold, which introduces larger errors in the gradient components of these small molecules.

**Table 3:** Average percentage of retained NAFs calculated with different ε_NAF_ thresholds (in a.u.) for Baker’s test set

ε_NAF_	cc-pVDZ	aug-cc-pVDZ	cc-pVTZ
max	25	32	38
10^–2^	36	32	44
5 · 10^–3^	40	36	51
10^–3^	54	51	67
10^–4^	68	70	85
10^–5^	82	85	95
10^–6^	94	96	98

**Figure 4:**
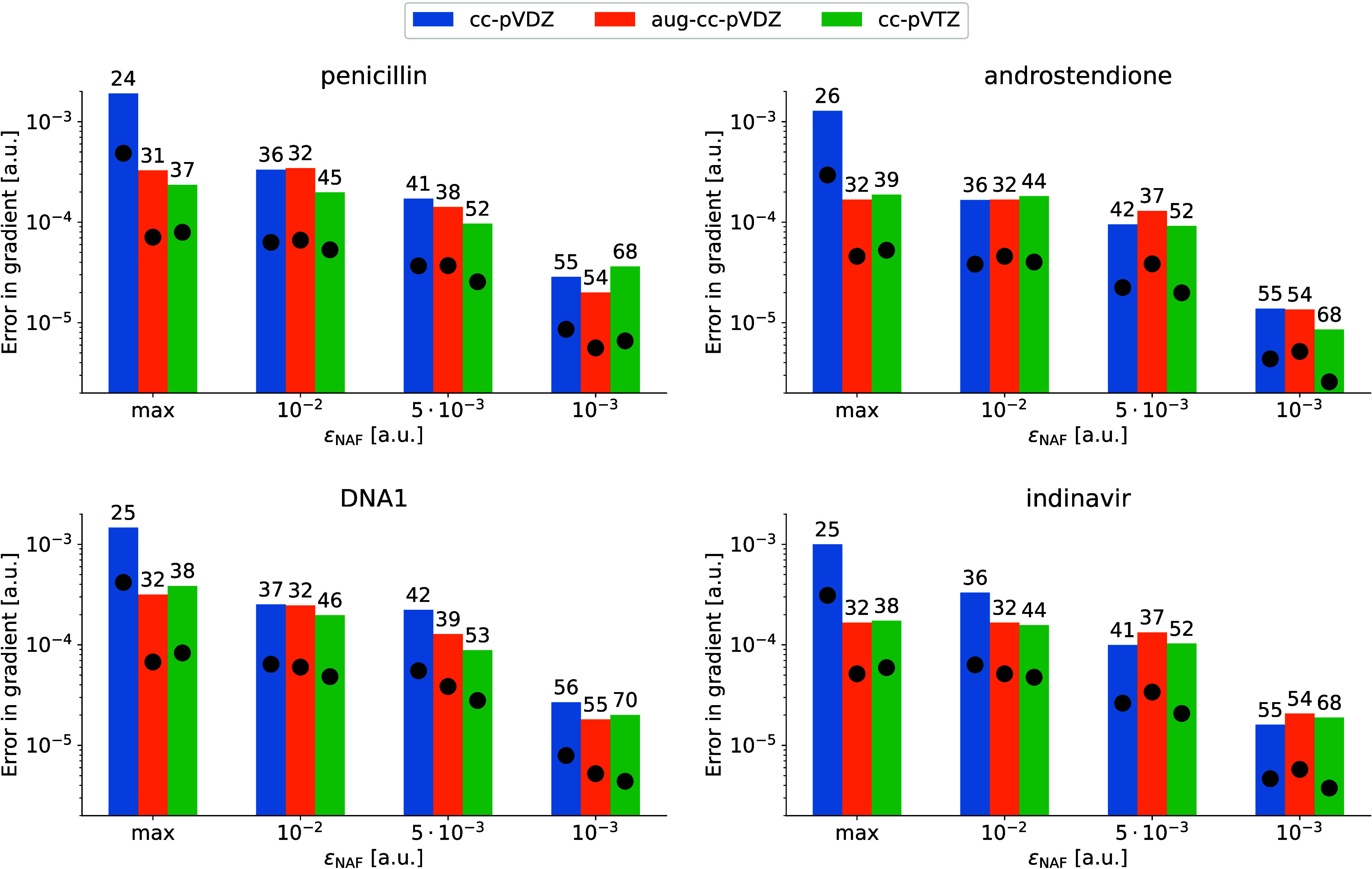
Error of NAF-MP2 gradients as a function of ε_NAF_ calculated for selected test molecules with three different basis sets. The bars and dots correspond to maximum errors and rms errors, respectively. Numbers on the top of the bars denote the relative number of kept NAFs in %.

The gradient errors of several larger molecules also support the above findings. Figure 4 shows the max and rms errors of the gradients for the penicillin, androstendione, DNA1, and indinavir molecules along with the relative number of retained NAFs above the error bars. Similarity in the nature of the plot and in the actual number of kept NAFs compared to the corresponding plots for the small molecules is conspicuous. Thus, we can draw the conclusion that, independently of the system size, keeping almost the minimal number of the NAFs with ε_NAF_ = 10^–2^ a.u. guarantees sufficient accuracy in the energy gradient. However, using exactly the minimal number of NAFs, that is ε_NAF_ = max, can introduce accidental errors of 1 order of magnitude larger with small basis sets. Thus, we propose ε_NAF_ = 10^–2^ a.u. as the default value for the eigenvalue threshold.

### Smoothness of Potential Energy Surfaces

**Figure 5:**
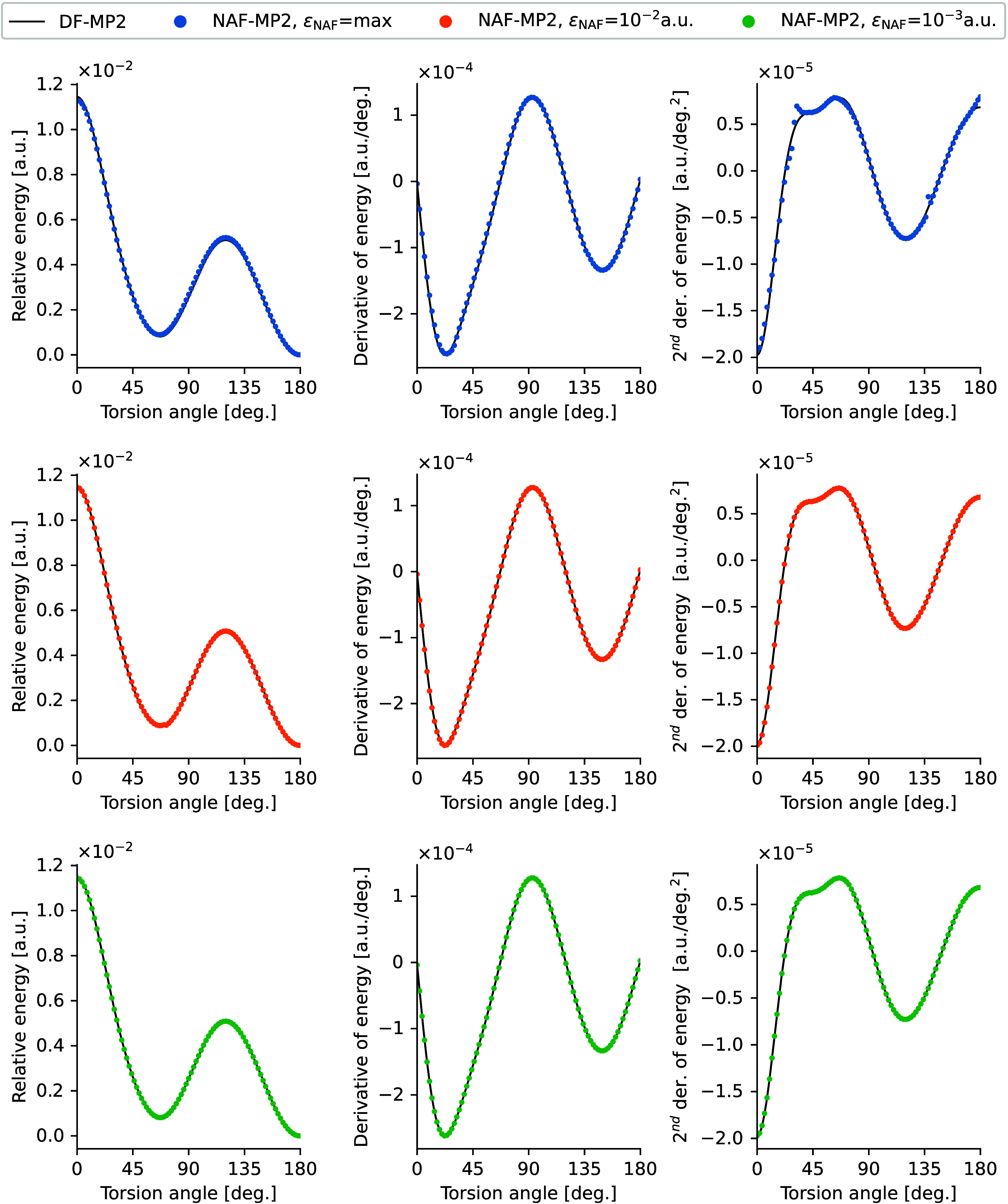
Potential energy surface, analytic first derivative, and numerical second derivative (with step size of 0.05°) for the central C–C torsion of C_10_H_22_ calculated with NAF-MP2 using various ε_NAF_ thresholds and with DF-MP2.

We tested the smoothness of the potential energy surface (PES) on the example of the torsional PES of C_10_H_22_ with ε_NAF_ = max, 10^–2^, and 10^–3^ a.u. with the cc-pVDZ basis compared to the corresponding DF-MP2 curves.^101^ We rotated the two fragments of the molecule around the central C–C bond and plotted the potential energy relative to the all-trans conformer (180° torsional angle), the analytic first derivative of the energy, and the numerical second derivative of the energy calculated from the analytic gradient (with 0.05° step size) in Figure 5. The PES along with the numerical first and second derivatives can be seen in Figure S1 of the Supporting Information. The maximum error of the relative energy is 10^–4^, 7 · 10^–5^, and 7 · 10^–6^ a.u. for ε_NAF_ = max, 10^–2^, and 10^–3^ a.u., respectively. The first derivative of the energy, calculated with various ε_NAF_ thresholds, closely matches the curve obtained using the parent DF-MP2 method, with maximum errors of 9 · 10^–6^, 2 · 10^–6^, and 9 · 10^–8^ a.u./deg. for ε_NAF_ = max, 10^–2^, and 10^–3^ a.u., respectively. The numerical second derivative of the energy on the PES closely follows the DF-MP2 reference curve, deviating from it only for ε_NAF_ = max at critical torsional angles. The maximum errors amount to 2 · 10^–6^, 2 · 10^–7^, and 8 · 10^–8^a.u./deg.^2^, respectively.

**Figure 6:**
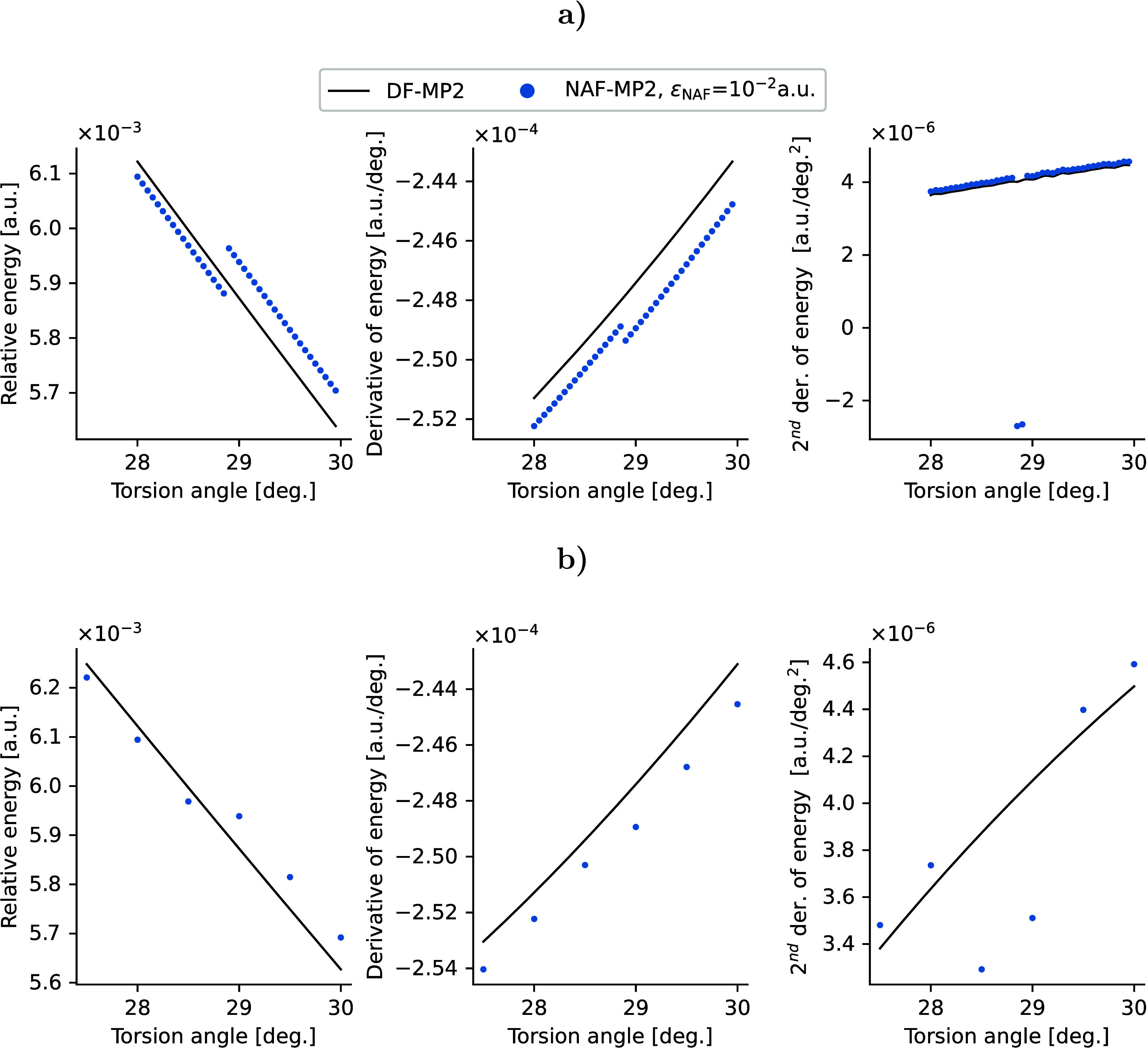
Potential energy surface, analytic first derivative, and numerical second derivative for the central C–C torsion of C_10_H_22_. The step size for the numerical differentiation is a) 0.05°, b) 0.5°.

The number of kept NAFs is constant for the ε_NAF_ = max threshold (*n*_NAF_ = *n*_AO_ = 240, *n*_aux_ = 868), however, it can change along the PES for other values of ε_NAF_. For ε_NAF_ = 10^–2^ and 10^–3^ a.u., the number of kept NAFs changes only by ±1 for extensive regions of the PES around *n*_NAF_ = 305 and 475, respectively, as shown in Figure S2 of the Supporting Information. Examining these regions more closely on the example of ε_NAF_ = 10^–2^ in the range of 28–30° plotted in Figure 6, we observe minor discontinuities on the PES and in the analytic derivative when the number of NAFs steps +1. This jump in energy and gradient is within the range of the error of these values compared to the DF-MP2 results. Nevertheless, the discontinuity in the first derivative of the energy causes significant changes in the numerical second derivative. As the jump in the first derivative is rather small, this issue can be addressed by increasing the step size in the numerical differentiation. Changing the step size of the numerical differentiation from 0.05° to 0.5° reduces the jump to 6 · 10^–7^ a.u. almost within the maximum error range of the second derivative compared to DF-MP2.

In summary, the proposed default threshold of ε_NAF_ gives appropriately smooth PES and analytic first derivative of the PES to calculate quantitatively correct numerical second derivatives.

### Efficiency of NAF-MP2

**Figure 7:**
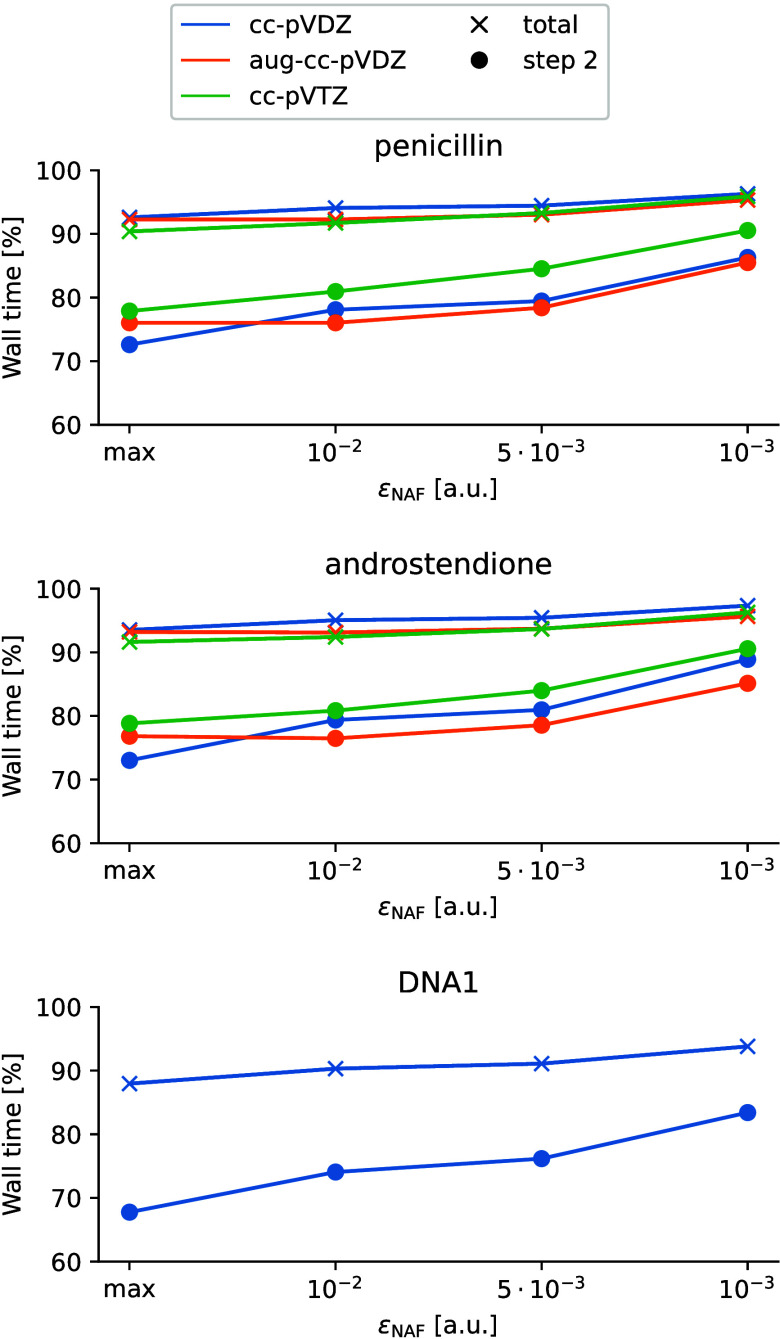
Relative wall time with respect to standard DF-MP2 of the NAF-MP2 gradient calculation and step 2 of the algorithm as a function of ε_NAF_ calculated with three different basis sets. The calculations for the selected molecules and basis sets ran with one correlated occupied MO batch.

To evaluate the efficiency of the NAF-MP2 code, we measured the overall wall time and the wall time of step 2 of the algorithm in comparison to the standard DF-MP2 method. Figure 7 shows the molecule and basis set combinations that did not take advantage of the batching structure of our algorithm, that is, the rate-limiting intermediates could be kept in the main memory and did not need to be split into smaller blocks. In these cases both the NAF-MP2 and the DF-MP2 code run in one correlated occupied batch of MOs, thus the algorithm of step 2 saves several I/O and matrix operations. In that system size range, step 2 is not the rate-determining step of the overall calculation, rather the solution of the Z-vector equation (step 3) means the most time-consuming part of the algorithm (see SI).

It is apparent from the plots that increasing ε_NAF_ smoothly increases the relative computation times of the NAF-MP2 gradient calculation. The results also suggest that the molecular size highly affects both wall time values. With increasing molecular size, the relative wall time of NAF-MP2 drops systematically. The time requirement of step 2 shows increasing character with the size of the basis set despite the slight decrease of the total time. This is caused by the fact that with increasing system size, the weight of step 2 in the total time grows as well. Thus, the minor time gain in step 2 has higher effect in the total time. For the default value, ε_NAF_ = 10^–2^ a.u., the time requirement of step 2 ranges from 70% to 80%. However, the overall computation time is also influenced by other parts of the algorithm, and 5–10% decrease in the overall wall time is expected for systems of that size.

**Figure 8:**
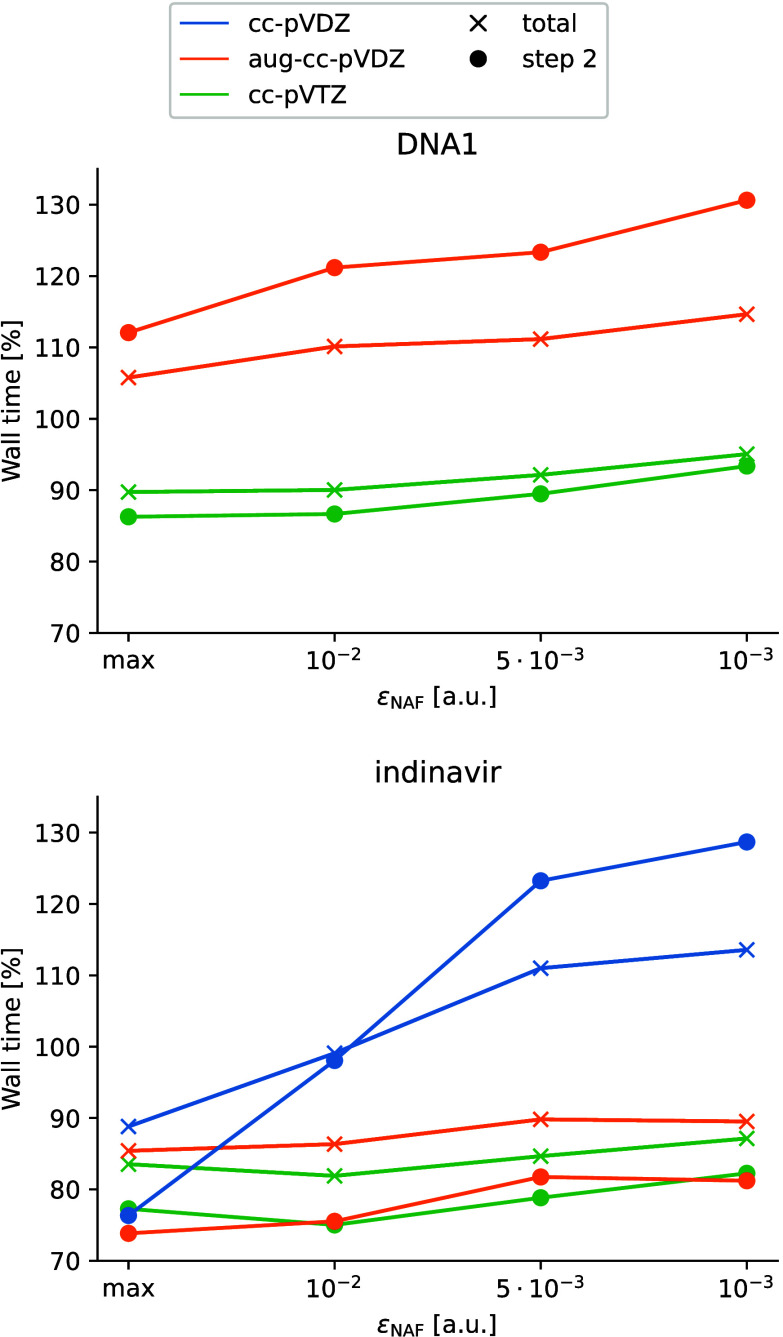
Relative wall time with respect to standard DF-MP2 of the NAF-MP2 gradient calculation and step 2 of the algorithm as a function of ε_NAF_ calculated with three different basis sets. The calculations for the selected molecules and basis sets ran with more than one correlated occupied MO batch.

Figure 8 shows molecule and basis set combinations where step 2 of the DF-MP2 and NAF-MP2 algorithms is executed with two or more batches. For the smallest system of this kind, DNA1 with aug-cc-pVDZ basis set, the NAF-MP2 algorithm looses its efficiency compared to DF-MP2. This is a consequence of the fact that the NAF-MP2 algorithm contains several additional loops for correlated occupied batches of MOs with respect to DF-MP2. These additional I/O and matrix operations initially overcome the gain in the prefactor of the fifth-power scaling operations with NAF-MP2. However, increasing the size of the molecule or the basis set, the NAF approximation reduces the time requirement of step 2. In this system size range, step 2 becomes the most time-consuming step in the calculation, thus the total time-reduction is more and more determined by the time requirement of step 2. For ε_NAF_ = 10^–2^ a.u., the relative computation time of step 2 approaches 75%, and the overall wall time decreases to 80% for systems such as indinavir with the cc-pVTZ basis set.

## Conclusions

In this work, we presented the derivation of analytic first derivatives for the NAF-MP2 method in attempt to decrease the size of the auxiliary basis set required for the density fitting. An efficient implementation of the approach was reported and used to benchmark the accuracy and efficiency of the NAF approximation in gradient calculations and geometry optimizations of small and medium-sized test molecules.

The errors in the calculated gradients and optimized geometric parameters were found to be sufficiently small. Setting the truncation thresholds to its default value, ε_NAF_ = 10^–2^ a.u., the NAF approximation results in rms errors of smaller than 10^–4^ a.u. in the calculated gradient elements, while the typical errors in bond lengths (angles) are around 10^–4^ Å (10^–4^ deg.).

The smoothness of the potential energy surface was tested on the torsional PES of C_10_H_22_. The PES and the analytic first derivative of the PES were found to be appropriately smooth with maximum errors of 7 · 10^–5^ a.u. and 2 · 10^–6^ a.u./deg., respectively, calculated with the default threshold of ε_NAF_ = 10^–2^ a.u. These properties enable the calculation of quantitatively correct numerical second derivatives with a maximum error of 2 · 10^–7^a.u./deg.^2^

The computational costs of step 2 of the algorithm can be reduced to around 70% for systems calculated in one batch (e.g., with our computer hardware, the DNA1 molecule with cc-pVDZ basis set). However, the overall time requirement is shadowed by other parts of the algorithm, thus a decrease of 5–10% in the total wall time is expected in these cases. For larger systems that run in more batches, the computational expenses of step 2 and the overall algorithm smoothly decrease with the system size, and a gain of around 20% is achieved in the case of the indinavir molecule with the cc-pVTZ basis set.

All in all, the reported NAF-MP2 analytic gradients allow for calculating accurate gradients compared to the parent DF-MP2, though with moderate time-reduction. However, the derivation presented here also serves as an important step toward the analytic gradients of NAF-based higher-level correlation methods, such as CCSD(T), where significantly higher gains are envisaged.

## Supporting Information Available

Potential energy surfaces (PDF)
Number of atoms, electrons, AOs, fitting functions, and computation times for our test systems (XLS)
