# Supplementary material for: Analytic Gradients for Density Fitting MP2 Using Natural Auxiliary Functions
Source: J Phys Chem A. 2024 Jul 29;128(31):6566–80. doi: 10.1021/acs.jpca.4c02822 (PMC11317987; doi:10.1021/acs.jpca.4c02822)
Supplement: Supplementary file 1 — jp4c02822_si_001.pdf [file jp4c02822_si_001.pdf]

Supporting Information for "Analytic gradients for density fitting MP2 using natural auxiliary functions"

Klára Petrov,[*,†,‡,¶] József Csóka,[*,†,‡,¶] and Mihály Kállay[*,†,‡,¶]

†*Department of Physical Chemistry and Materials Science, Faculty of Chemical Technology and Biotechnology, Budapest University of Technology and Economics, Műegyetem rkp. 3., H-1111 Budapest, Hungary*

‡*HUN-REN–BME Quantum Chemistry Research Group, Műegyetem rkp. 3., H-1111 Budapest, Hungary*

¶*MTA–BME Lendület Quantum Chemistry Research Group, Műegyetem rkp. 3., H-1111 Budapest, Hungary*

E-mail: klara.petrov@gmail.com; csoka.jozsef94@gmail.com; kallay.mihaly@vbk.bme.hu

Z-matrix of $C_{10}H_{22}$ structures for the calculation of the torsional PES:

c
c 1 1.53951
c 1 1.53951 2 113.458
c 2 1.53951 1 113.458 3 dih0
h 2 1.10097 1 109.238 3 dih1
h 2 1.10097 1 109.238 3 dih2
h 1 1.10097 2 109.238 4 dih1
h 1 1.10097 2 109.238 4 dih2
c 3 1.53951 1 113.461 2 180.000
h 3 1.10097 1 109.236 2 57.870
h 3 1.10097 1 109.235 2 -57.87
c 4 1.53951 2 113.461 1 180.000
h 4 1.10097 2 109.235 1 57.869
h 4 1.10097 2 109.235 1 -57.87
c 9 1.53951 3 113.454 1 180.000
h 9 1.10097 3 109.239 1 -57.87
h 9 1.10097 3 109.239 1 57.872
c 12 1.53951 4 113.454 2 -180.00
h 12 1.10097 4 109.239 2 -57.87
h 12 1.10097 4 109.239 2 57.873
c 15 1.53951 9 113.464 3 -180.00
h 15 1.10097 9 109.235 3 -57.87
h 15 1.10097 9 109.235 3 57.870
c 18 1.53951 12 113.465 4 180.000
h 18 1.10097 12 109.235 4 -57.87
h 18 1.10097 12 109.235 4 57.869
h 21 1.10097 15 109.239 9 180.000
h 21 1.10097 15 109.239 9 -57.87
h 21 1.10097 15 109.240 9 57.873
h 24 1.10097 18 109.240 12 -180.00
h 24 1.10097 18 109.240 12 57.873
h 24 1.10097 18 109.240 12 -57.87

where dih0 is the torsion angle varied in the range of $0°$ to $180°$,
dih1 = -121.806 + dih0,
dih2 = 122.452 + dih0

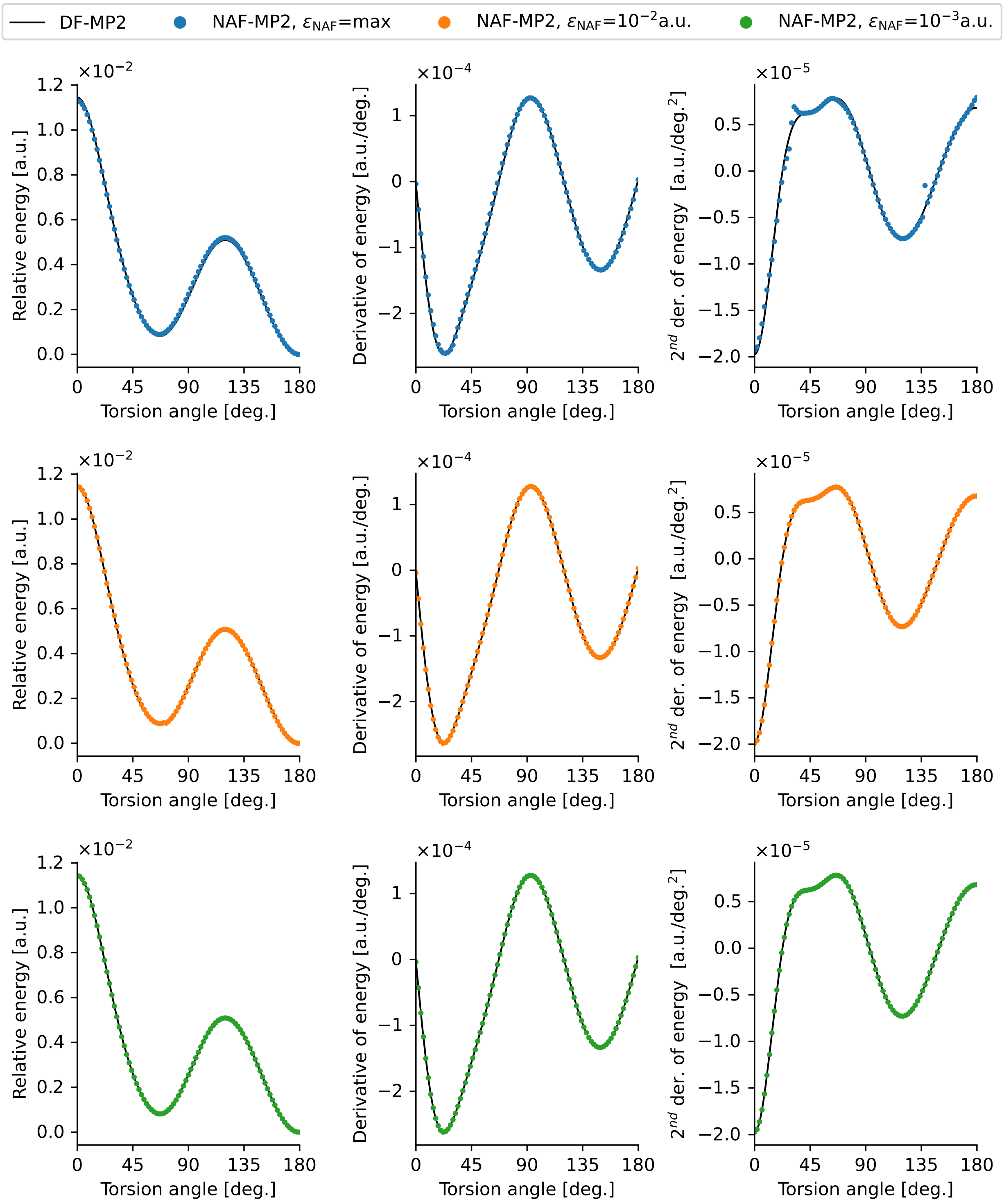

Figure S1: Potential energy surface, numerical first and second derivative (with 0.05° step size for the numerical differentiation) of the PES for the central C–C torsion of $C_{10}H_{22}$ calculated with NAF-MP2 using various ε_{NAF} thresholds and with DF-MP2.

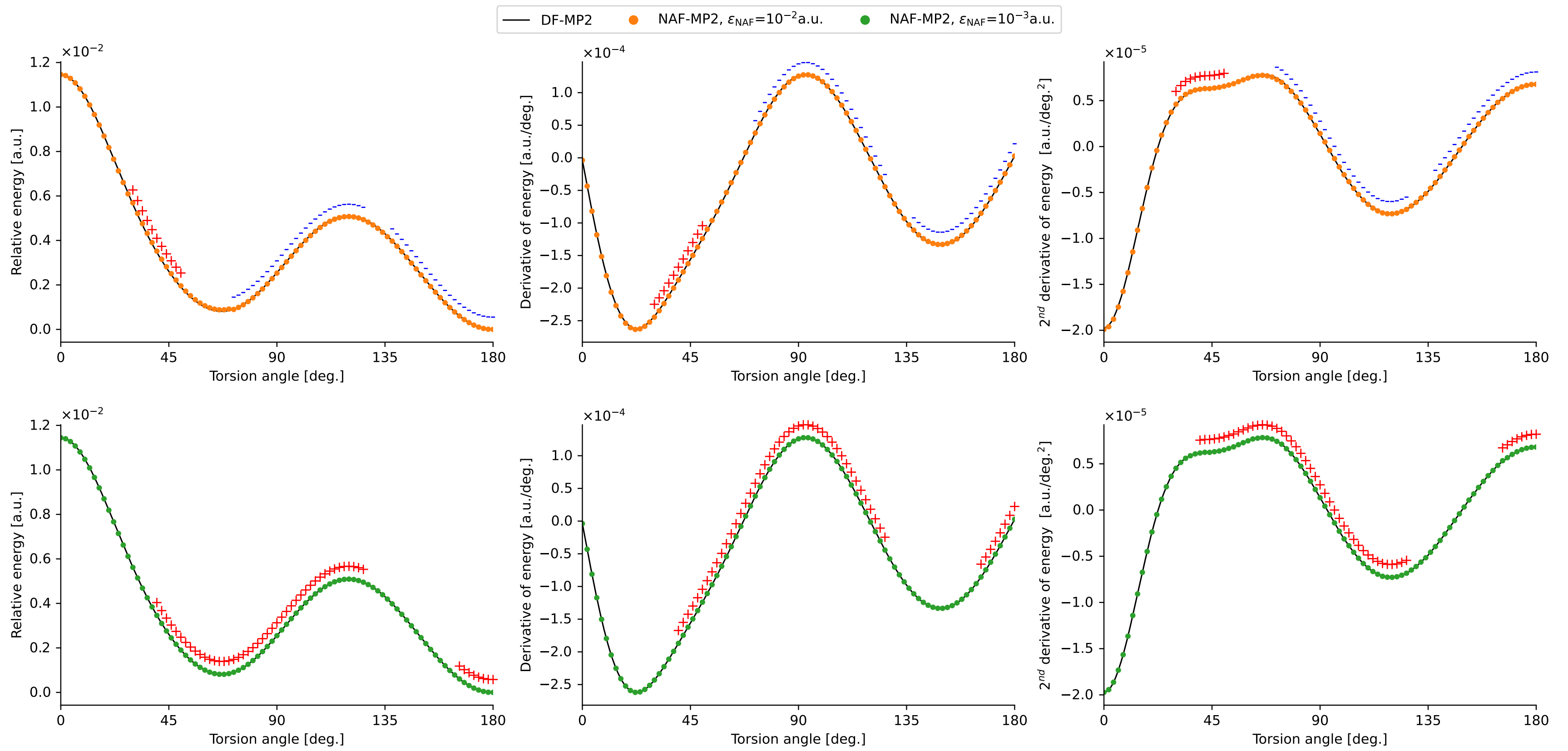

Figure S2: Potential energy surface, analytic first derivative, and numerical second derivative of the PES for the central C–C torsion of $C_{10}H_{22}$ calculated with NAF-MP2 using various $\varepsilon_{\mathrm{NAF}}$ thresholds and with DF-MP2. The plus and minus signs above the points indicate the regions of the PES where the number of kept NAFs changes by +1 or −1, respectively.
